# Chronic Sublethal Effects of Cantharidin on the Diamondback Moth *Plutella xylostella* (Lepidoptera: Plutellidae)

**DOI:** 10.3390/toxins7061962

**Published:** 2015-05-29

**Authors:** Zhengyu Huang, Yalin Zhang

**Affiliations:** Key Laboratory of Plant Protection Resources and Pest Management of the Ministry of Education, College of Plant Protection, Northwest A&F University, Yangling 712100, Shaanxi, China; E-Mail: huangzy0503@163.com

**Keywords:** sublethal effects, multigeneration, susceptibility, demographic parameters, Integrated pest management

## Abstract

The diamondback moth, *Plutella xylostella* (Linnaeus) (Lepidoptera: Plutellidae), is a major pest of cruciferous vegetables worldwide. Cantharidin, a natural toxin isolated from blister beetles, has been reported to be toxic to *P. xylostella*. However, little is known on the chronic sublethal effects of cantharidin on this species. In this study, we assessed the changes of susceptibility, development, reproduction and other demographic parameters in both the selected *P. xylostella* strain (Sub, selected by LC_25_ cantharidin for consecutive 12 generations) and the revertant strain (SubR, derived from the Sub strain without being exposed to cantharidin for 12 generations). Results revealed that the two strains maintained a relatively high-level susceptibility to cantharidin. Severe adverse effects on the population dynamics and fitness in Sub strain were observed. In addition, repeated exposure of *P. xylostella* to sublethal concentration of cantharidin resulted in negative effects on adult performance and deformities in adults. Although morphologically normal for individuals, the SubR strain exhibited a disadvantage in population growth rate. Our results showed that sublethal concentration of cantharidin exhibited severe negative effects on population growth for longtime. These findings would be useful for assessing the potential effects and risk of cantharidin on *P. xylostella* and for developing effective integrated pest management.

## 1. Introduction

The diamondback moth, *Plutella xylostella* (L.), is regarded as the most destructive pest of cruciferous vegetables throughout the world due to its high rate of consumption, high fecundity, and short generation time (up to 20 generations per year) [1,2,3]. Currently, its control is not always adequate, probably owing to the development of multiple and cross-resistance to nearly all groups of pesticides applied in the field such as organophosphates, carbamates, pyrethroids, diacylhydrazines, spinosyns and *Bacillus thuringiensis*, *etc.* [4].

The natural toxicant cantharidin, an active constituent of blister beetles, has been found to be toxic to some pests [5,6,7,8]. Recently, advances in toxicology are changing the way we evaluate toxicants [9]. Besides direct mortality, exposure to a toxicant can result in multiple sublethal effects on population dynamics through impairment to physiological traits, such as: life span [10], development rate [11], fecundity [12,13], and egg hatchability [14]. These effects can provide a comprehensive understanding and prediction of the total effects of a toxicant at the population level [9,15,16].

Recent studies demonstrated that a sublethal concentration of cantharidin significantly affected the development and reproduction of insects including *M. separate*, *H. armigera* and *P. xylostella* [17,18,19]. However, these studies only covered a period of a few days, leaving us without knowledge of the effects of chronic exposure of this toxicant on insects. The chronic exposure studies mimicked environmentally realistic conditions and monitored the effects of repeated exposure to toxicants over long time periods. Some studies have demonstrated that repeated exposure to sublethal concentrations of toxicants may lead to lower susceptibility [20] or enhanced population growth [21,22]. In addition, the insects can recover their biological characteristics and fitness along with restoring susceptibility when the toxicants are removed [23]. These changes could be major factors in recurrent pest outbreaks and resurgences [22,24,25,26]. Therefore, sublethal long-term effects analysis of toxicants, estimating the total effect of insecticides on populations, is crucial when choosing new pesticides for controlling pests.

In this study, the stability of susceptibility was investigated in both a cantharidin-selected strain (Sub strain) and a revertant strain (SubR strain). In addition, chronic experiments were conducted to assess serial changes of development, reproduction and other demographic parameters for *P. xylostella* in the presence or absence of cantharidin exposure. We aim to investigate the sublethal effects of cantharidin on *P. xylostella* over multiple generations and determine how they changed with time in the presence or absence of this stressor.

## 2. Results

### 2.1. Susceptibility of SS, Sub and SubR Strains to Cantharidin

The 48 h LC_25_ of cantharidin for the cantharidin-susceptible strain (SS strain) was 3.84 mg·L^−1^, and this concentration was chosen for a selection experiment (Table 1). After being selected for 12 generations, the LC_50_ value of the Sub-12 was higher than that of the SS, but their 95% confidence interval (CI) values overlapped, suggesting that their LC_50_ values were not significantly different (*p <* 0.05). The slope of the regression curve increased 31.76%, indicating that the individuals of the sub strain increased in homogeneity when selection cycles were increased. In the first two revertant generations, the LC_50_ value of cantharidin declined from 18.24 mg·L^−1^ to about 11.00 mg·L^−1^ in the absence of exposure. The LC_50_ value was maintained at similar level in the 12th revertant generation (Table 1).

**Table 1:** Susceptibility of SS (cantharidin-susceptible strain), Sub (cantharidin-selected strain) and SubR (revertant strain) strains of *Plutella xylostella* to cantharidin (48 h).

Strain	N ^a^	Slope ± SE	LC_50_ (95% confidence interval) (mg·L^−1^)	χ^2^	Toxicity ratio ^b^	LC_25_ (95% confidence interval) (mg·L^−1^)
SS ^c^	320	1.48 ± 0.17	10.95 (8.35–15.01)	4.01	1.00	3.84 (2.73–5.07)
Sub-6 ^d^	320	1.50 ± 0.17	11.34 (8.66–15.57)	4.09	1.04	4.02 (2.87–5.29)
Sub-12 ^e^	320	1.90 ± 0.20	18.24 (14.51–23.64)	5.11	1.67	8.04 (6.08–10.16)
SubR-1 ^f^	320	1.59 ± 0.18	12.11 (9.35–16.41)	1.98	1.07	4.57 (3.33–5.95)
SubR-2 ^g^	320	1.53 ± 0.17	10.22 (7.87–13.77)	3.25	1.03	3.71 (2.65–4.87)
SubR-6 ^h^	320	1.63 ± 0.17	8.89 (6.95–11.68)	4.69	0.81	3.43 (2.49–4.45)
SubR-12 ^i^	320	1.56 ± 0.17	11.01 (9.35–16.41)	3.59	1.05	4.06 (2.93–5.31)

^a^ Number of larvae tested; ^b^ LC_50_ of selected strain/LC_50_ of SS strain; ^c^ SS, cantharidin-susceptible strain; ^d^ Sub-6, LC_25_ cantharidin-selected SS strain for 6 generations; ^e^ Sub-12, LC_25_ cantharidin-selected SS strain for 12 generations; ^f^ SubR-1, a revertant strain, derived from a substrain of Sub-12 which had not been exposed to cantharidin or any other insecticides for one generation; ^g^ SubR-2, a revertant strain, derived from a substrain of Sub-12 which had not been exposed to cantharidin or any other insecticides for two consecutive generations; ^h^ SubR-6, a revertant strain, derived from a substrain of Sub-12 which had not been exposed to cantharidin or any other insecticides for 6 consecutive generations; ^i^ SubR-12, a revertant strain, derived from a substrain of Sub-12 which had not been exposed to cantharidin or any other insecticides for 12 consecutive generations.

### 2.2. Effects of Cantharidin on Life History of Different Strains

The developmental durations of the Sub and SubR strains changed across generations (Table 2). Negative effects were observed in Sub strain. Development of the immature stages was decelerated in both sexes compared to the SS strain. This decelerated development of immature stages primarily appeared in the extension of larval and pupal periods. These effects were significant in the first generation, but gradually faded in latter generations. Additionally, when treated with cantharidin, the adult longevity of females was remarkably reduced in the Sub strain. Cantharidin also significantly influenced the duration of the adult total preoviposition period (TPOP) of *P. xylostella* in Sub-1 and Sub-6. However, the sublethal effects on Sub-12 were diminished and exhibited no significant difference in comparison to the SS strain, except for the pupal period and adult longevity of females. SubR strain showed a similar duration of whole development in comparison to the SS strain. The developmental durations of eggs were prolonged in both Sub and SubR strains, but the difference from that of the SS strain was not significant (Table 2).

**Table 2:** The effects of cantharidin on development (means ± SE) of *Plutella xylostella* in different strains.

Strain	Life stage								
	Egg	Larva	Pupa	Duration of immature stage		Adult longevity		APOP ^1^	TPOP ^2^
				Female	Male	Female	Male	Female	Female
SS	3.29 ± 0.02 a	7.59 ± 0.04 c	4.21 ± 0.02 c	15.51 ± 0.07 bc	14.80 ± 0.11 c	13.34 ± 0.21 a	14.01 ± 0.28 a	0.59 ± 0.06 b	16.09 ± 0.12 c
Sub-1	3.33 ± 0.05 a	8.49 ± 0.08 a	4.58 ± 0.04 a	16.84 ± 0.13 a	16.03 ± 0.12 a	9.77 ± 0.36 d	11.93 ± 0.30 b	0.93 ± 0.05 a	17.77 ± 0.14 a
Sub-6	3.45 ± 0.07 a	7.97 ± 0.09 b	4.33 ± 0.06 bc	15.93 ± 0.10 b	15.57 ± 0.09 b	10.90 ± 0.24 cd	13.01 ± 0.53 a	0.86 ± 0.06 ab	16.79 ± 0.11 b
Sub-12	3.39 ± 0.02 a	7.67 ± 0.04 c	4.42 ± 0.02 ab	15.52 ± 0.10 bc	15.21 ± 0.13 bc	11.90 ± 0.31 bc	13.50 ± 0.19 a	0.74 ± 0.09 ab	16.26 ± 0.05 c
SubR-12	3.38 ± 0.02 a	7.71 ± 0.05 bc	4.25 ± 0.03 c	15.31 ± 0.11 c	15.30 ± 0.08 b	12.33 ± 0.27 ab	13.95 ± 0.23 a	0.70 ± 0.09 ab	16.00 ± 0.17 c
*p*	0.084	<0.0001	<0.0001	<0.0001	<0.0001	<0.0001	0.001	0.018	<0.0001
*F*	2.329	33.766	14.452	34.460	18.045	11.910	6.765	3.648	35.313
*df*	4,25	4,25	4,25	4,25	4,25	4,25	4,25	4,25	4,25

Means marked with different letters within the same column are significantly different (*p* < 0.05; Tukey’s HSD test). ^1^ APOP, adult preoviposition period, time between adult emergence and first oviposition; ^2^ TPOP, total preoviposition period, time from birth to first reproduction in female.

### 2.3. Effects of Cantharidin on Adult Performance and Reproductive Potential of Different Strains

Cantharidin exhibited a significant effect on the reproductive parameters of female adults (Table 3). The oviposition period in both Sub and SubR strains tended to be reduced, even though this reduction gradually became not significant with the increase in selected generations. The reproductive potential investigation mainly included fecundity (total number of eggs laid per female), fertility (the percentage of eggs hatched) and egg size. It is worth noting that the egg size of Sub and SubR strain was obviously different from SS strains (Table 3). The eggs from the Sub strain, especially in the 1st generation, were much smaller. Thereafter, this size recovered slightly in the 6th generation and fluctuated at around 1.23 × 10^−2^ mm^3^ in the following generations. The Sub strain accumulated a decrease in fertility accompanying the incremental selection generations. The fertility in Sub-12 was significantly decreased by 11.00%. However, the fertility in SubR-12 recovered but was still significantly lower than in the SS strain. Compared with the SS strain, the reproductive effort per female was significantly lower in Sub-1 and Sub-6. However, there was no difference found among the Sub-12 and SubR-12 cohorts (Table 3).

**Table 3:** The effects of cantharidin on adult fitness and reproduction potential (means ± SE) of *Plutella xylostella* in different strains

Strain	Fecundity ^1^	Egg Size (10^−2^ mm^3^) ^2^	Reproductive effort per female ^3^	Oviposition duration in days	Fertility (% egg hatch)
SS	203.97 ± 3.40 ab	1.32 ± 0.02 a	2.69 ± 0.06 a	8.37 ± 0.15 a	86.86 ± 0.75 a
Sub-1	161.26 ± 2.43 c	1.21 ± 0.02 b	1.96 ± 0.05 c	6.95 ± 0.17 b	85.80 ± 0.86 ab
Sub-6	187.41 ± 5.67 b	1.24 ± 0.02 b	2.33 ± 0.10 b	7.18 ± 0.17 b	83.46 ± 1.10 ab
Sub-12	217.15 ± 7.87 a	1.22 ± 0.02 b	2.64 ± 0.08 a	7.79 ± 0.25 ab	75.86 ± 1.44 c
SubR-12	195.38 ± 4.99 ab	1.25 ± 0.02 b	2.43 ± 0.07 ab	7.89 ± 0.16 a	82.34 ± 0.52 b
*p*	<0.0001	0.001	<0.0001	<0.0001	<0.0001
*F*	18.752	6.214	15.939	9.624	19.175
*df*	4,25	4,25	4,25	4,25	4,25

Means marked with different letters within the same column are significantly different (*p* < 0.05; Tukey’s HSD test); ^1^ Total number of eggs laid by each female; ^2^ Eggs laid on the first oviposition day; ^3^ Fecundity × egg size.

### 2.4. Effects of Cantharidin on Age-Stage Survival Rates (s_xj_) of Different Strains

**Figure 1:**
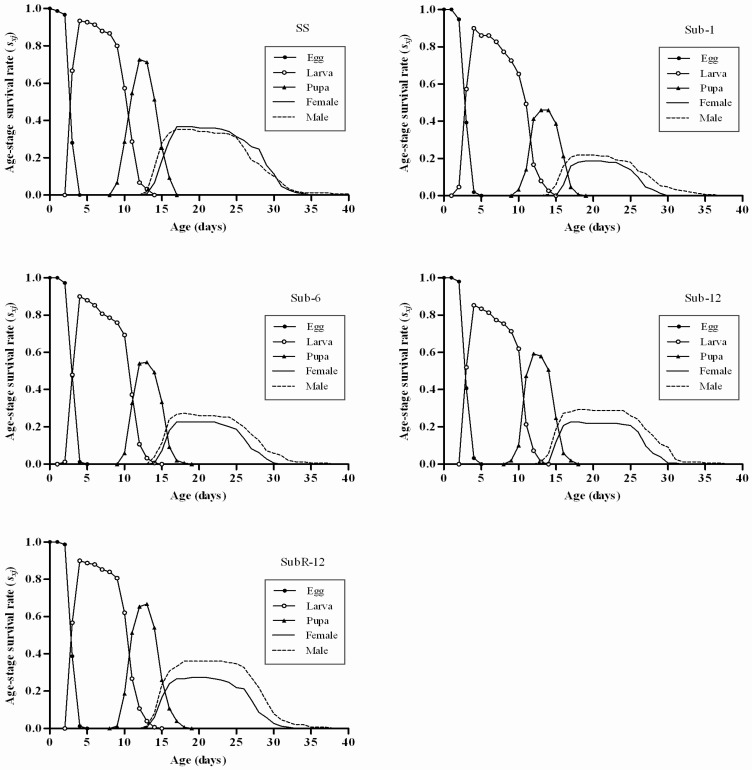
Age-stage survival rates (*s_xj_*) of *Plutella xylostella* in different strains.

Lower survival curves in all stages were observed in the Sub strains (Figure 1). It was noticeable that in Sub-1 and Sub-6, the maximum values of survival rate at larval and pupal stages were less than those of the SS.

In addition, negative effects of cantharidin were observed during the adult stage for both sexes. Under sublethal-selected conditions, both females and males exhibited shorter survival periods and lower survival rates. However, these negative effects diminished with the increase in selection generations. Survival rates of the SubR strain at the immature stage were similar to that of the SS strain.

### 2.5. Effects of Cantharidin on Age-Specific Survival Rate (l_x_), Age-Specific Fecundity (m_x_) and Age-Specific Maternity (l_x_m_x_) of Different Strains

**Figure 2:**
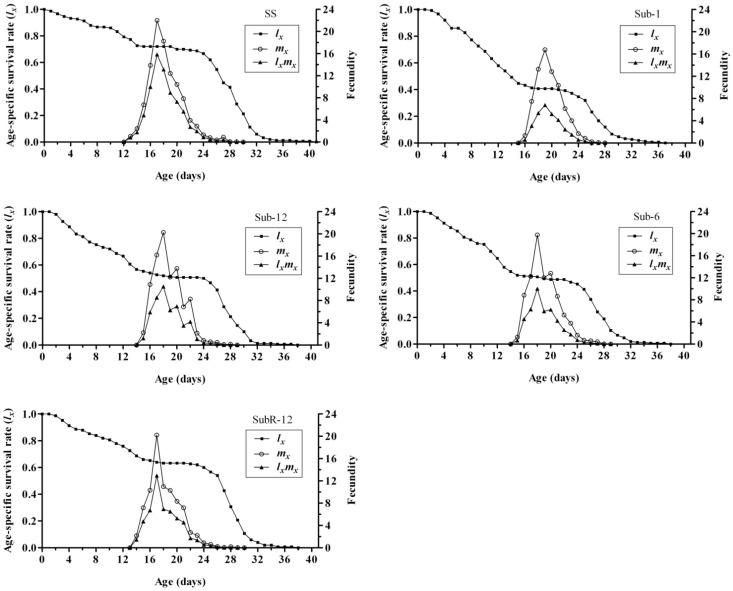
Age-specific survival rate (*l_x_*), age-specific fecundity (*m_x_*) and age-specific maternity (*l_x_m_x_*) of *Plutella xylostella* in different strains.

The *l_x_* of the Sub strain was also negatively affected by cantharidin (Figure 2). However, the results showed *P. xylostella* survived and reproduced successfully in all strains. The sublethal cantharidin treatment did not interrupt the life cycle of *P. xylostella*. In the SS strain, *l_x_* decreased gradually along the whole life course of *P. xylostella*. The trends of *l_x_* were similar between the Sub and SS strains. Along with the increase of selection generations, *l_x_* was reverted in comparison with that of SS. Mortalities of SubR and SS strains were similar throughout the whole life course.

When taking fertility into consideration, the negative effect of cantharidin was evident in age-specific fecundity (*m_x_*) (Figure 2). In SS, females began to oviposit on day 13 and the age-specific fecundity rapidly reached a peak at 22.02 eggs per female on day 17, followed by a continual fall until death. Sub, SubR and SS strains possessed a similar trend in the age-specific fecundity. However, peak values in the former two strains were lower. The initial time of oviposition in different cohorts (Sub-1, Sub-6, Sub-12, SubR-12), on the 16th, 15th, 15th and 14th day respectively, lagged behind. The cohorts from Sub strain (Sub-1, Sub-6, Sub-12) reached their climax on day 19th, 18th, and 18th, respectively, with peak time being later than that of SS (Figure 2).

### 2.6. Effects of Cantharidin on Demographic Parameters and Fitness

Comparison of various demographic parameters among the SS, Sub and SubR strains revealed significant differences that might be correlated with the population fitness (Table 4). In different cohorts (Sub-1, Sub-6, Sub-12, SubR-12), the demographic parameters of *P. xylostella* including net reproduction rate (*R*_0_), intrinsic rate of increase (*r*), finite rate of increase (*λ*) and gross reproduction rate (GRR) tended to be lower than those of the SS. In addition, mean generation time (*T*) was prolonged. Sublethal effects of cantharidin on the Sub strain tended to decline with the increase in selection cycles. Clearly, there were significant differences in all mentioned parameters between the SS and Sub strains except for GRR. In absence of the cantharidin selection, the population characteristics in the SubR strain had recovered to some degree when compared with those of the Sub strain. The rate of relative fitness reflected a recovery in the SubR strain as well (Table 4). However, the SubR strain still exhibited a serious disadvantage in population growth rate compared to the SS strain including decreased *R*_0_, *r* and *λ*.

**Table 4:** Sublethal effects of cantharidin on the demographic parameters (means ± SE) in different *Plutella xylostella* strains

Strain	Parameter					
	*R*_0_	*r*	*λ*	GRR	*T* (d)	Rate of relative fitness ^1^
SS	74.80 ± 5.94 a	0.23 ± 0.01 a	1.26 ± 0.01 a	105.25 ± 7.36 a	18.59 ± 0.15 c	1.00
Sub-1	30.15 ± 3.67 c	0.18 ± 0.01 d	1.18 ± 0.01 d	75.17 ± 7.49 b	20.34 ± 0.13 a	0.40
Sub-6	43.40 ± 4.91 b	0.19 ± 0.01 c	1.21 ± 0.01 c	87.80 ± 8.52 ab	19.55 ± 0.13 b	0.58
Sub-12	49.08 ± 5.47 b	0.20 ± 0.01 c	1.22 ± 0.01 c	94.95 ± 9.17 ab	19.30 ± 0.09 b	0.66
SubR-12	53.41 ± 5.09 b	0.21 ± 0.01 b	1.24 ± 0.01 b	83.68 ± 7.10 b	18.59 ± 0.14 c	0.71

Means marked with different letters within the same column are significantly different (*p* < 0.05; Tukey-Kramer test); ^1^ *R*_0_ of selected strain/*R*_0_ of SS strain.

### 2.7. Abnormalities Caused by Cantharidin Treatment

**Figure 3:**
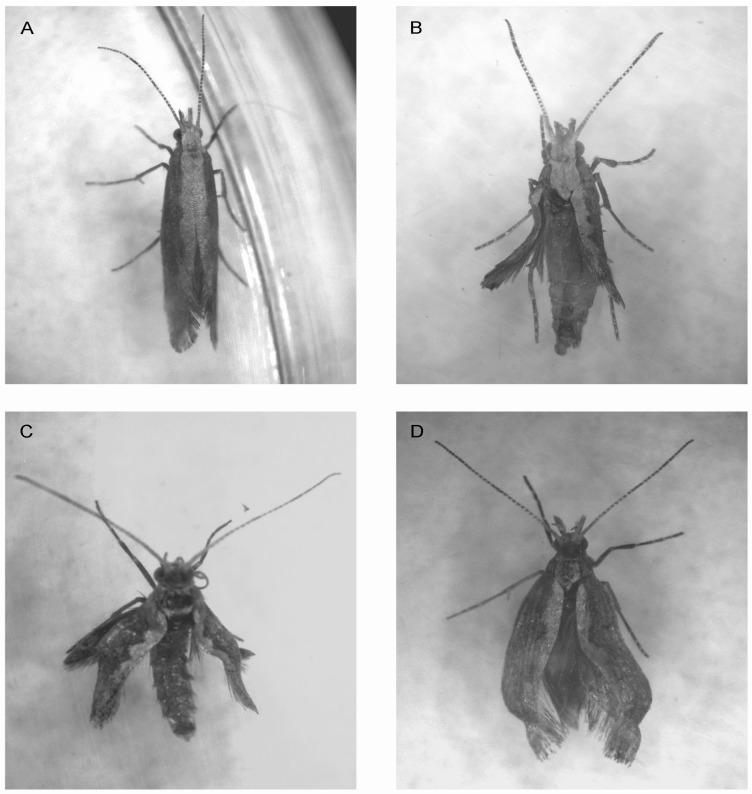
Various morphological abnormalities in treated *Plutella xylostella* with sublethal concentration of cantharidin. (**A**) normal adult; (**B**) miniature crippled wings in adult; (**C**,**D**) twisted wings in adults.

**Figure 4:**
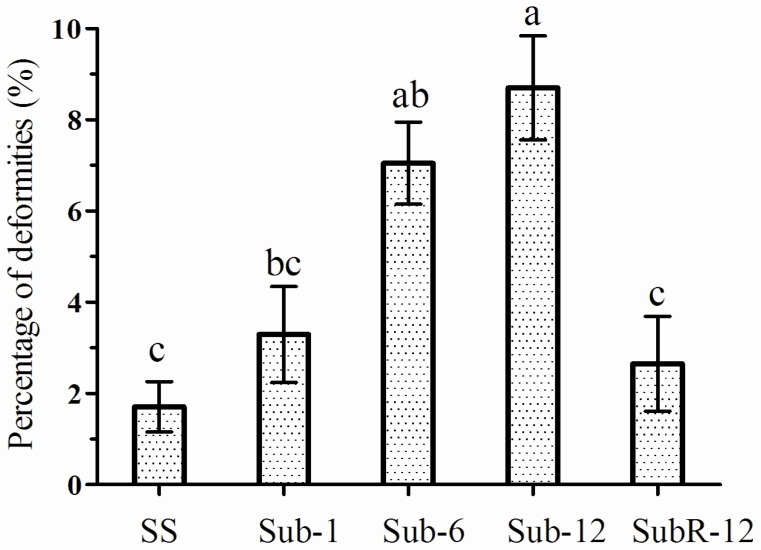
Percentage of abnormalities in different *Plutella xylostella* strains. The results are presented as means (±SE). Histograms bearing different letters are significantly different at the *p* < 0.05. (Tukey’s HSD test).

Malformation and abnormalities of adults were noticed in the cantharidin treatment (Figure 3) and this progressively became more apparent as the number of sublethal selection cycles increased (Figure 4).

## 3. Discussion

In this study, we found cantharidin maintained a high toxicity in the selected strain (Sub). The susceptibility of the third instar larvae of *P. xylostella* to cantharidin decreased slightly as the number of sublethal selection cycles increased. Similar results were found in *P. xylostella* treated with sublethal concentration of spinosad [22]. However, such a reduction can be mostly recovered in less than two generations when the selection pressure is eliminated. The SubR strain maintained a stable and high susceptibility similar to the SS strain throughout the following generations.

The selection experiment in our study showed that frequent application of low-dose cantharidin may change the sublethal effects of cantharidin on *P. xylostella* with time. In general, the sublethal effects on Sub-1 were significant, and these effects were mainly expressed in the differing biological characteristics and demographic parameters when compared with the SS strain. These negative effects still existed in the Sub strain after being selected for 12 generations, although most biological characteristics recovered gradually.

The egg incubation period was not significantly affected whereas larval and pupal development was decelerated strongly in Sub-1 and Sub-6 cohorts. Our finding is consistent with previous reports of short-term exposure [17,18,19]. A similar negative effect in larval and pupal development of *P. xylostella* was also observed after being exposed to sublethal concentrations of hexaflumuron [27]. A further harmful effect of cantharidin was observed on adult performance of *P. xylostella*. In the current study, fecundity was significantly reduced in Sub-1. Similar phenomena were also found in *P. xylostella* treated with azadirachtin and neemarin [28,29]. Such reduction on fecundity by sublethal concentrations of insecticides may contribute to depressing future population growth [30]. Previous research suggested that the reduction in fecundity might be related to physiological and morphological changes in females of different orders [31]. Therefore, a sublethal concentration of cantharidin could significantly influence the reproductive capacity of females. However, these effects gradually faded in latter generations. In the present study, the fecundity of the Sub-12 was slightly higher than that of the SS strain, but no mathematically significant difference was observed. Similar result concerning changes in fecundity of *Chironomus riparius* Meigen exposed to 159.6nM Cd was observed, wherein the fecundity gradually recovered after a sharp decrease in the first three generations [32]. One possible reason for this finding may be that the surviving females allocate a specific amount of nutrition across more eggs to maintain high reproductive rates and ensure population stability in the future [33].

*P. xylostella* eggs became smaller in the first generation under LC_25_ selection and this smaller size was maintained thereafter. This indicated that selection under cantharidin favors a diminished egg size in *P. xylostella*. The smaller egg size in the Sub strain may be a parallel phenomenon caused by selection at sublethal doses of cantharidin. A similar phenomenon was found in *P. xylostella* that treated with fenvalerate [34]. Moreover, in the Sub strain, the survival rate of larvae hatched from the smaller eggs was lower than that from normal-sized eggs in the SS strain. Yin *et al.* also reported that the survival females laid smaller eggs after the third instar larvae were treated with the LC_25_ concentration of spinosad, and the smaller eggs showed a lower hatchability [22]. This may be due to the fact that the embryo failed to complete the development phase or perforated the surrounding vitelline membrane [35]. In general, a smaller insect egg results in lower viability in the subsequent developmental stages [36,37,38]. Females of the SubR strain also laid fewer and smaller eggs, even though they were not exposed to the cantharidin for 12 generations. This suggests that smaller-sized eggs may be a genetic trait and selection by cantharidin produces a diminished egg size in *P. xylostella* [34]. These results suggest that the SubR strain also has a lower fitness level in the absence of selection pressure.

The demographic toxicological analysis that incorporates life table parameters has been recommended for evaluating the effect of an insecticide [39,40]. In this study, the analyses of estimated demographic parameters in the Sub strain provided insights into the adverse effects of sublethal cantharidin on *P. xylostella* on a generational scale. *R*_0_, *r* and *λ* significantly decreased during the first generation (Sub-1). These all imply an adverse impact on population growth in *P. xylostella*. In addition, *T* tended to be longer, and the extended *T* may cause a remarkable deceleration of life cycle and fewer generations per year. By reference to previous studies, insects treated with the sublethal concentration of the same insecticide might exhibit differential effects along with the time of exposure. Vogt *et al.* reported that the population growth rate was remarkably decreased in the first generation when *C. riparius* were exposed to low concentrations of tributyltin but for generations seven to ten, the population growth rate tended to recover [21]; Yin *et al.* demonstrated that the population growth rate was significantly decreased in *P. xylostella* in the first generation against a LC_25_ dose of spinosad; after five to ten generations, however, the population growth rate gradually recovered [22]. Similar results concerning changes in demographic parameters were observed in our multigenerational experiment. For the following generations in Sub strain, all above-mentioned parameters and the population relative fitness recovered gradually. This may be due to the development of physiological adaptation of *P. xylostella* to sublethal concentration of cantharidin [41]. Although these effects of demographic parameters can be recovered when the selection pressure is removed, the SubR strain still maintained a disadvantage in relative fitness (0.71) even after 12 generations. Decreased demographic parameters and fitness indicated a profound reduction in the forthcoming generation, thus maintaining the pest population below a level of economic loss, this is of practical importance [42].

Additionally, twisted and crippled wings were observed among both males and females. This could cause flight disability and mating disruption. Similar symptoms were found in previous studies in our lab for *H. armigera* and the decreased activity of alkaline phosphatase (ALP) was considered as a factor causing these deformities [18]. An earlier study demonstrated that the activity of ALP was significantly lower after cantharidin treatment, and this decrease became more noticeable with the extension of exposure time [6]. We also observed that the changes in morphological traits became more obvious as the number of sublethal selection cycles increased. This showed that cantharidin may take effect over a long period, accumulating damaging sublethal effects later in life.

## 4. Materials and Methods

### 4.1. Insects and Chemicals

An insecticide-susceptible strain of *P. xylostella* was maintained in our laboratory (Key Lab of Plant Protection Resources & Pest Management of Ministry of Education, Northwest A&F University, Yangling, China) for over five years without exposure to any insecticide. Larvae were reared on vermiculite-cultured pakchoi (*Brassica chinensis* L.) seedlings at 25 ± 2 °C, 50% ± 5% relative humidity (RH) with a photoperiod of 16:8 (L:D) h. Adults were provided with 10% honey solution as nutrient.

Cantharidin used in this study was extracted from *Mylabris phalerata* Pallas (Chinese blister beetle) (purity > 98%) and stored in our lab.

### 4.2. Bioassay

A leaf-spray bioassay method [43] was used to determine the susceptibility of *P. xylostella* to cantharidin. Compounds were dissolved in a component solvent (water:acetone = 19:1, *v*/*v*) containing 0.5% Tween-80 (Shanghai Jingchun Reagent Co., Shanghai, China) as emulsifier. On the basis of preliminary assays, at least seven serial dilutions ranging from 0.625 to 60 mg·L^−1^, were used. Bioactivity against the healthy third instar larvae was tested using treated cotyledons of pakchoi seedlings [44]. The third instar larvae were identified by head capsule width [45,46]. Cotyledons, were sprayed evenly by test solutions for about 5s using a chromatographic sprayer [43], air dried for about 25 min at room temperature and transferred to Petri dishes (9 cm diameter) lined with a slightly moistened filter paper. Four replicates of ten larvae were used per concentration. Treatment with solvent was carried out as the control. Larvae were starved for 4 h and then released in Petri dishes with ten treated cotyledons. Subsequently, larvae were kept in a growth cabinet (ZPQ-280, Dongtuo Instrument Co. LTD, Harbin, China) under the standard conditions mentioned above, and mortality was recorded after 48 h.

### 4.3. Selection Experiments

Based on the bioassay results, 48 h LC_25_ concentration was used for the subsequent experiments. The Sub strain (selected by LC_25_ cantharidin for every generation) was consecutively selected by using the leaf-spray method. Strain Sub-n represents the strain selected for n generations consecutively. The third-instar larvae of each generation were fed with pakchoi cotyledons and treated with LC_25_ equivalent cantharidin for 48 h as described above. The larvae fed on the solvent treated cotyledons acted as the SS strain. The SS strain was reared in the laboratory in parallel with the Sub strain. The progeny of the selection-surviving insects were split into two groups: one for the next generation selection, and the other for toxicity testing. The revertant strain (SubR) was derived from a substrain of Sub-12 without exposure to cantharidin or any other insecticides. SubR-n represents the revertant strain without exposure to cantharidin or any other chemicals and insecticides for n generation(s). The Sub, SubR and SS strains were tested for their susceptibility to cantharidin (the concentration that kills, LC_50_) in each generation.

### 4.4. Biological Characteristics of Plutella xylostella in Different Strains

Demographic observations were conducted for the SS, Sub-1, Sub-6, Sub-12 and SubR-12 cohorts. For each colony, six replicates of fifty newly laid eggs were used. Each egg was placed in a Petri dish (9 cm diameter) and hatched under the same insect rearing conditions as described above. The hatched larvae were supplied with fresh pakchoi cotyledons that were changed when necessary. The egg incubation, larval, and pupal developmental periods and mortality were recorded every day until all larvae had either died or developed into adults. Following the emergence of adults, the abnormal ones were photographed and the percentage of abnormalities in adults was recorded. Moths that emerged on the same day were paired and allowed to lay eggs until the death of all individuals. The longevity of adults and the duration of pre-oviposition, oviposition and post-oviposition were recorded. In addition, the total number of eggs laid per female and the rates of hatching were recorded for the calculation of fecundity and fertility.

### 4.5. Egg Size

The sizes of 10 eggs laid by females on the first oviposition day from each replicate were measured using a stereo-microscope with an ocular micrometer (SMZ-1500, Nikon Co., Tokyo, Japan). The major axis (*a*) and minor axis (*b*) of the egg were measured, and the volume (*V*) was calculated using the formula *V =* π*ab*^2^/12, based on the assumption that the shape was half ellipsoid [47].

### 4.6. Age-Stage, Two-Sex Life Table Analysis

Life-history raw data from different strains were analyzed according to the age-stage, two-sex life table theory [48,49]. The age-stage specific survival rate (*s_xj_*) (where *x* is the age and *j* is the stage), the age-specific survival rate (*l_x_*) and age-specific fecundity (*m_x_*) were calculated. The *l_x_* and *m_x_* are estimated as:

(1) $$l_{x}=\sum_{j=1}^{k}s_{xj}$$

and

(2) $$m_{x}=\frac{\sum_{j=1}^{k}s_{xj}f_{xj}}{\sum_{j=1}^{k}s_{xj}}$$

The intrinsic rate of increase (*r*) is calculated iteratively from the Euler-Lotka equation with age indexing from 0 [50]:

(3) $$\sum_{x=0}^{\infty }e^{-r(x+1)}l_{x}m_{x}=1$$

The relationship between *R*_0_ and mean female fecundity, *F*, is defined as follows:

(4) $$R_{0}=F(N_{f})/N$$

where *N* is the total number of individuals used for life table study and *Nf* is the number of female adults [51]. The gross reproduction rate (GRR) is as follows:

(5) $$\text{GRR}=\sum_{x=0}^{\delta }m_{x}$$

where δ is the last age of the cohort. The mean generation time (*T*) is the length of time that a population needs to increase to *R*_0_-fold of its size (*i.e.*, *e^rT^ = R*_0_ or *λ^T^ = R*_0_) at the stable age-stage distribution. The *T* is calculated as:

(6) $$Τ=\frac{\text{ln}R_{0}}{r}$$

A computer program, TWOSEX-MSChart [51] was used for data analysis in Visual BASIC for the Windows operating system.

### 4.7. Data Analysis

Bioassay data were collected and analyzed by standard probit analysis, using Abbott’s correction [52] for control mortality. The LC_25_ and LC_50_ values were estimated from dosage-mortality regression by using the statistical program POLO-PC (LeOra Software Inc., Berkeley, CA, USA, 1987). The lethal concentration (LC) values for different strains were considered significantly different if their 95% confidence limits (CL) did not overlap. The sublethal effects of cantharidin on *P. xylostella* biological characteristics were analyzed by one-way ANOVA and Tukey’s HSD test (*p* < 0.05) using SPSS 12.0 (IBM Inc., Chicago, IL, USA, 2003). All data were checked for normality using a non-parametric Kolmogorov-Smirnov test (*p* < 0.05) and the fecundity data were log(*x* + 1) transformed, as necessary, but untransformed means were also presented. The means, variances and standard errors of the demographic parameters were estimated with the bootstrap procedure (B = 10,000) [53,54]. Mean values of these data were compared using the Tukey-Kramer procedure to admit significant differences at *p* < 0.05. The results of *s_xj_*, *l_x_*, *m_x_* and the percentage of deformities were plotted using GraphPad Prism 5 (GraphPad Software Inc., San Diego, CA, USA, 2007).

## 5. Conclusions

In summary, the consistency of high-level susceptibility of *P. xylostella* to cantharidin was observed through frequent application of cantharidin. Low-dose cantharidin exhibited severe negative effects on population growth throughout the Sub strain and even in the SubR-12, a revertant strain without selection by cantharidin for 12 consecutive generations. Taken together these results indicate that cantharidin serving as an effective alternative to conventional pesticides could be incorporated into integrated pest management program targeting *P. xylostella*.
